# Long-Term Changes in Organic Matter Content and Soil Moisture Determine the Degree of Root and Soil Respiration

**DOI:** 10.3390/plants12020251

**Published:** 2023-01-05

**Authors:** Zsolt Kotroczó, Marianna Makádi, Tamás Kocsis, Áron Béni, Gábor Várbíró, István Fekete

**Affiliations:** 1 Department of Agro-Environmental Studies, Hungarian University of Agriculture and Life Sciences, H-1118 Budapest, Hungary; 2 Research Institute of Nyíregyháza, University of Debrecen, H-4400 Nyíregyháza, Hungary; 3 Department of Food Microbiology, Hygiene, and Safety, Hungarian University of Agriculture and Life Sciences, H-1118 Budapest, Hungary; 4 Institute of Agricultural Chemistry and Soil Science, University of Debrecen, H-4400 Nyíregyháza, Hungary; 5 Department of Tisza River Research, Danube Research, Institute, Centre for Ecology of HAS, H-4026 Debrecen, Hungary; 6 Institute of Environmental Science, University of Nyíregyháza, H-4400 Nyíregyháza, Hungary

**Keywords:** CO_2_-emission, respiration components, litter manipulation, SOM, soil moisture

## Abstract

Carbon in soil is one of the most important indicators of soil fertility. Part of the carbon stored in them is returned to the atmosphere during soil respiration. Climate change and inappropriate land use can accelerate these processes. Our work aimed to determine how soil CO_2_ emissions change over ten years as a result of litter manipulation treatments. Plots at the Síkfőkút DIRT (Detritus Input and Removal Treatments) experimental site include doubling either leaf litter or wood, and removing all aboveground litter, all root inputs, or removing all litter inputs. With the help of this, we were able to examine not only the effects of the different organic matter intake but also the effects of the different microclimates that occur as a result of the treatments. Total soil respiration (root and microbial respiration) is a result of a persistent lack or excess of soil organic matter relative to soil moisture. Based on our studies, the increase in the intensity of root respiration on wetter soils was only half of the increase in respiration associated with decomposition activity. The sustained growth of leaf litter significantly increases soil respiration, which can be partly explained by the more favorable supply of nutrients to the decomposing organisms, and partly by the more favorable microclimatic conditions, however, these effects were only valid in the case of wetter soils. In the dry summer environment, we experienced higher CO_2_ emissions during litter removal treatments. In the first period between 2002 and 2004, even wetter root removal treatments showed a significantly higher CO_2_ emission, while in the period 2010–2012, surface litter removal treatments. The permanent removal of surface litter in the drier summer period resulted in the formation of a dense crack network, which increased the CO_2_ emission of these soils, which increases the soil organic carbon loss of the soil. Our study proves the advantages of mulching in terms of a more favorable microclimate of the soil surface and a balanced carbon balance of the soil–plant system.

## 1. Introduction

Soil organic matter (SOM) plays an extremely important role in the functioning and regulation processes of ecosystems. Soil organic matter is the ecosystem’s carbon (C) and nitrogen (N) reserve, which participates in cation exchange, soil pH control, and soil structure formation, and is the most important substrate for hetero-organotrophic soil microorganisms. The ability of the soil to store and accumulate carbon is also very significant in the global carbon cycle. Annually, from the decomposition of organic matter, approx. 9.4–9.8 × 10^16^ g of carbon leaves the soil, which is 2–3 times higher than the carbon content of the vegetation [1,2]. The emission of carbon from the soil is very intense, soil respiration is 10 times higher than the amount of CO_2_ from the burning of fossil fuels [2,3].

Soil respiration, the emission of CO_2_ from the soil to the atmosphere, is one of the most important components of terrestrial metabolism and is mainly the result of microbial degradation processes in the soil and respiration by plant roots [4,5]. The CO_2_ emissions of soil macro- and mesofauna are much smaller than the above, only a few percent [6]. CO_2_ gas exchange shows a high spatial and temporal variability in all ecosystems [7]; this is the key to adapting to ever-changing environmental conditions. Soil respiration is also an important part of the carbon turnover in forest ecosystems [8,9]. Large amounts of carbon are released into the atmosphere as carbon dioxide, mainly through the decomposition of above-ground litter (heterotrophic component) and CO_2_ emissions from living plant roots (autotrophic component) [5].

Soil respiration estimates have been made for many ecosystems, and several summaries have been produced [10,11]. Soil respiration can be separated into different components [12,13,14], but both autotrophic respiration (CO_2_ emissions from root respiration) and CO_2_ emissions from heterotrophs (CO_2_ from decomposition) are poorly understood and studied processes. The amount of carbon dioxide from above-ground decomposition can be directly estimated from the amount of soil carbon production [10]. However, the contribution of different soil carbon sources is difficult to calculate. For example, some estimates suggest that root respiration accounts for 3–5%, while others suggest that it may account for up to 60% [15,16].

Sources of CO_2_ emitted from soil can be identified in different ways [5]. Soil organic matter (SOM), above and below-ground dead plant remains (litter) and organic matter produced by living roots (root exudates, exudates, secretions, detached root cells), can be separated based on soil and surface carbon stocks. Based on the location of CO_2_ production, sources can be distinguished as root-free soil, root-permeated soil (rhizosphere), and litter layer. The presence of growing roots, as well as the litter level, plays an extremely important role in soil CO_2_ emissions [8].

Soil carbon, which is on average almost double the carbon stored in biomass in forests [17], can also be affected by human activities: deforestation and soil disturbance can significantly accelerate the decomposition of soil organic matter and increase the risk of erosion [18,19]. The intensity of soil respiration is a basis for soil quality assessment and, together with bacterial biomass and enzyme activity studies, can help to reveal the consequences of many physical, biological, and chemical changes affecting soils.

The Detritus Input and Removal Treatment (DIRT), a multi-decade litter manipulation field experiment [20], investigates how litter production changes under climate change and how artificially altering litter input (organic matter input) affects soil organic matter dynamics, biological activity, or soil respiration. Our work aimed to assess the changes in litter input after more than ten years of artificial changes. We aimed to determine, using soil respiration values of different treatments, how soil CO_2_ emissions change and how the distribution of the different components of total soil respiration (root respiration, respiration resulting from microbial decomposition of fresh litter) is affected by the persistent lack or surplus of organic matter concerning soil moisture content.

## 2. Results and Discussion

### 2.1. Changes in Soil Respiration during Dry Periods

**Figure 1:**
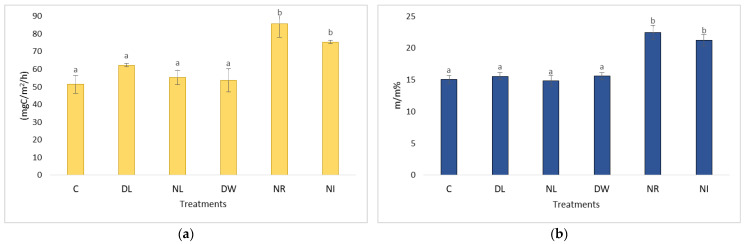
The value of the C content of soil respiration (**a**) and soil moisture (**b**) after the establishment of the experimental plots in the dry period (2002–2004). The letters represent significant differences between treatments during the given period (*n* = 6). C = control; DL = Double Litter; NL = No Litter; DW = Double Wood; NR = No Roots; NI = No Inputs.

A few years after the establishment of the plots, during the drier periods, the withdrawal (NR and NI) treatments showed the highest CO_2_ emissions (Figure 1). This was because the vegetation (trees, shrubs) was cut in the plots of these treatments. Many fresh organic plant remains (mainly root remains of various sizes) remained in the soils. The decomposition of these remains increased the rate of soil respiration. In the first few years, especially during the drier periods, the effect of this process was evident, because due to the lack of living vegetation, the amount of evapotranspiration on the NR and NI plots was much lower, and transpiration practically ceased on these plots and the litter cover in the NR plot prevented evaporation from the soil surface. Soil moisture decreased among treatments in the following order: NR (33.5%), NI (30%), DW (24.8%), C (24.1%), DL (23.7%), and NL (22%) [21,22]. Thus, the soil moisture of these significantly exceeded the values measured for the soils of the other treatments, and the breakdown of the roots in the soil was more intense in the wetter soils (Figure 1).

**Figure 2:**
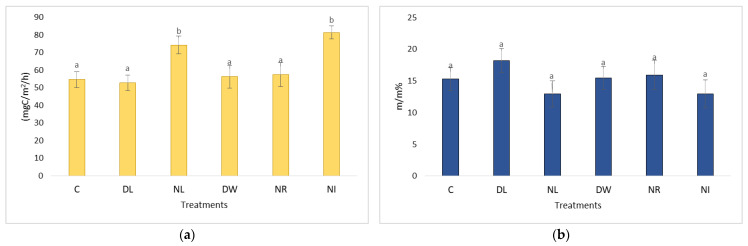
The value of the C content of soil respiration (**a**) and soil moisture (**b**) 10 years after the establishment of the experimental plots in the dry period (2010–2012). The letters represent significant differences between treatments during the given period (*n* = 6). C = control; DL = Double Litter; NL = No Litter; DW = Double Wood; NR = No Roots; NI = No Inputs.

Based on Fekete et al.’s [21] previous studies, they reached a similar conclusion. Based on their test results, the removal of aboveground vegetation determines the number of organic residues that can be found in the soil for years. The dead root mass left in the soil takes several years to decompose. In the second measurement period (between 2010 and 2012), the situation changed significantly (Figure 2). Due to the lack of carbon supply from litterfall and living root systems, the carbon content of the soils of litter removal treatments has decreased over the years [22].

### 2.2. Evolution of Soil Carbon Content at the End of the Experimental Period

**Figure 3:**
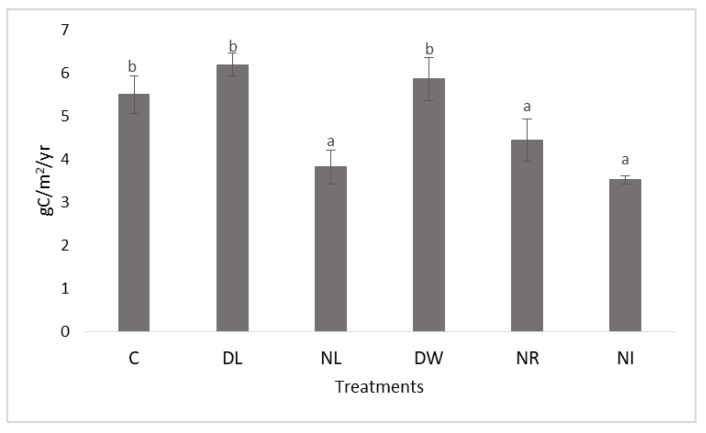
The average carbon content of soil at the end of the examined period (2013). The letters represent significant differences between treatments during the given period (*n* = 6). C = control; DL = Double Litter; NL = No Litter; DW = Double Wood; NR = No Roots; NI = No Inputs.

**Figure 4:**
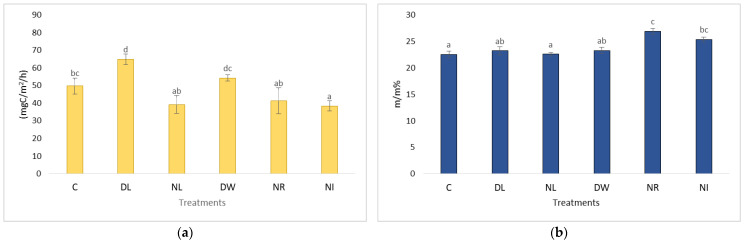
The value of the C content of soil respiration (**a**) and soil moisture (**b**) after the establishment of the experimental plots in the wet period (2002–2004). The letters represent significant differences between treatments during the given period (*n* = 6). C = control; DL = Double Litter; NL = No Litter; DW = Double Wood; NR = No Roots; NI = No Inputs.

**Figure 5:**
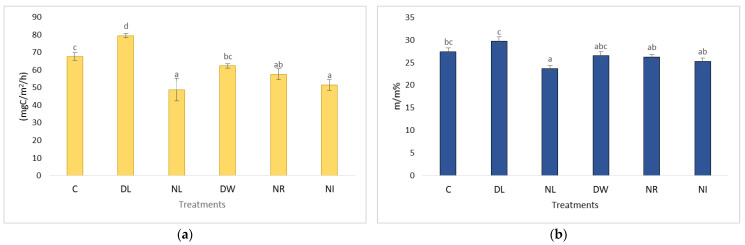
The value of the C content of soil respiration (**a**) and soil moisture (**b**) 10 years after the establishment of the experimental plots in the wet period (2010–2012). The letters represent significant differences between treatments during the given period (*n* = 6). C = control; DL = Double Litter; NL = No Litter; DW = Double Wood; NR = No Roots; NI = No Inputs.

As a result, after the second measurement cycle (2013), these plots had significantly lower carbon content than the litter-doubled and control plots (Figure 3). The soils of the plots with the lowest organic matter content and no surface litter layer (NL and NI) dried out the best during the second measurement period. Due to the lower organic matter and moisture content, the cracking of the soils increased in the dry summer months. As a result of the deep cracks, significantly more oxygen reached the deeper, wetter soil layers, thereby accelerating the decomposition of organic matter in the deeper soil layers as well. The formed CO_2_ could reach the surface more easily through the cracks, increasing the CO_2_ emission of these treatments (Figure 4 and Figure 5). Thus, the remarkable situation arose that we measured significantly higher CO_2_ emissions in the treatment with the lowest organic matter and thus the driest surface, free of surface litter, than in all other treatments. However, this finding was only true for the dry summer measurement period having a moisture content value lower than 20%. 

### 2.3. Changes in Soil Respiration during Dry Periods

In the wetter periods, the No Litter and No Input treatments showed the lowest soil respiration values, which were significantly lower compared to the Control, Double Wood, and Double Litter treatments (Figure 4 and Figure 5).

Due to the lower organic matter content and the lack of surface litter cover, the water storage capacity of these soils was significantly limited [22], therefore we measured the lowest average moisture content among the treatments in the second measurement period (2010–2012). We experienced this even in the case of the No Input treatment, which had the second-highest soil moisture in the first measurement period (after the establishment of the plots). Despite the absence of transpiration in this treatment, the two opposite effects mentioned were more significant. 

### 2.4. The CO*_2_* Emissions of the Treatments Withdrawal (NL, NR, NI) and Doubling (DL, DW) and C in the Case of Soil Moisture below 20 m/m%

**Figure 6:**
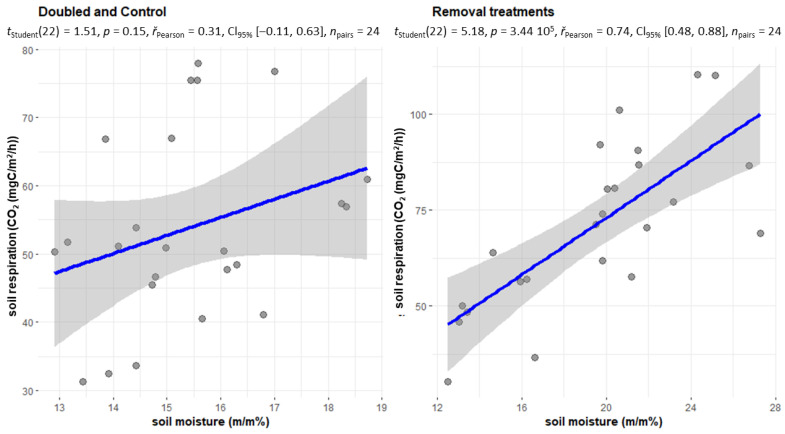
In the period 2002–2004, CO_2_ emissions of the two groups of treatments (removal (NL, NR, NI) and doubling (DL, DW) and C treatments) in the case of soil moisture below 20 *m*/*m*%.

Soil respiration is significantly influenced by temperature values [23,24]. Furthermore, the supply of nutrients to the soil microorganisms is also a determining factor [21,25], which in this case is influenced by the litter production dictated by the treatments. In the initial period between 2002 and 2004, doubled treatments (DL, DW) showed a slightly higher value in humid conditions compared to the dry period. In the case of the control treatment, a slightly (3%) higher value was also observed in the dry period, while the intensity of soil respiration was significantly higher in the dry period in the litter withdrawal treatments. The dry periods are primarily associated with the hottest summer months since transpiration is significant during this period, which also contributed to the drying of the soils of the treatments with living vegetation. The soils of the No Root and No Input treatments were also significantly wetter during the summer than the soils of the other treatments. This explains why soil respiration in these two treatments in the dry period is twice as great as in the wet (but cooler) periods. Furthermore, there is no litter cover on the soil surface of the No Input and No Litter treatments, therefore the cooling is much more significant here in the winter period, the number of frosty days is much higher [21], so the value of soil respiration is much lower here in the wet and partially cold periods. The temperature of the soil is much more even on the Control and Double Wood plots, and especially on the Double Litter plots. The period between 2010 and 2012 showed partially similar values for the DL and DW treatments. Even the Control plot showed higher values in the wet period. In the No Root treatment, soil respiration was balanced in the two periods, but in the case of NL and NI, soil respiration was much higher in the dry period than in the wet period. In the case of (dry) soils with a moisture content of less than 20% in the period 2002–2004, litter removal treatments showed a strong correlation with soil moisture (r = 0.74). We used the soil moisture of the Control treatment as a basis, if it was below 20%, the conditions were considered dry (Figure 6). 

**Figure 7:**
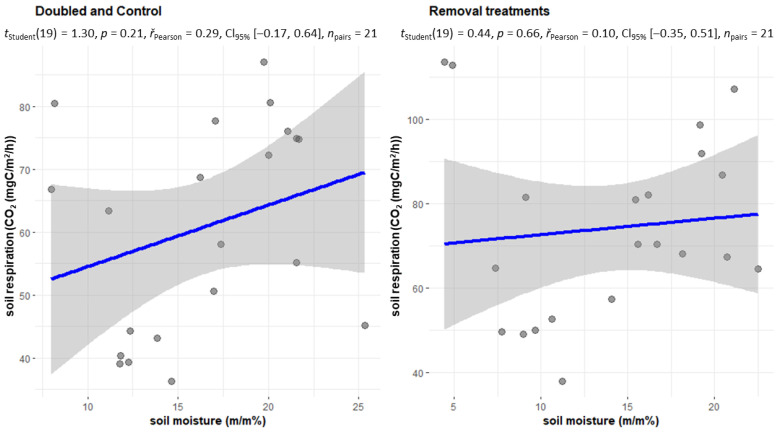
In the period 2010–2012, CO_2_ emissions of the two groups of treatments (removal (NL, NR, NI) and doubling (DL, DW) and C treatments) in the case of soil moisture below 20 *m*/*m*%.

In the treatments with litter removal (especially in the NR and NI treatments without transpiration), the average soil moisture was significantly higher (NR = 22.5%, NI = 21.2%, while C = 15.5%) than in the other treatments. Thus, the increase in moisture content in this region significantly increased the decomposition of organic matter, which was available in large quantities due to the mass of dead root litter. Furthermore, in the initial years of the experiments, the soil of the NR and NI treatments had a high SOC content, so the decomposition of these organic substances could also be higher due to the wetter conditions. In the period 2010–2012, we were no longer able to show any correlation in the case of withdrawal treatments either (Figure 7).

On the one hand, the reason for this is that the water retention capacity of the soils (NL, NR, NI), which were depleted in organic matter in the meantime, also declined significantly, so the moisture content of the NI (and NL) treatment was significantly lower than that of the Control plot. The NR treatment was approximately the same as the Control and less than the DL treatment. Furthermore, for the NL and NI treatments, there was also a negative correlation when the soil cracked during dry periods, thus increasing CO_2_ emissions. Probably, in the case of very dry soils, a smaller increase in moisture does not cause a significant increase in soil respiration, while it does in a slightly higher moisture range. In wet soils, the temperature is the main regulating factor, above a certain moisture limit, water displaces soil air from the soil pores, which inhibits aerobic decomposition processes, so heterotrophic soil respiration also declines. From the perspective of soil CO_2_ emissions, soil moisture is an important environmental factor that can affect different components of soil respiration in different ways [26]. Respiration resulting from microbial decomposition primarily responded to soil moisture and temperature, while root respiration was more indirectly related to photosynthetically active radiation (PAR) according to research results conducted in more sensitive grasslands [27]. In studies carried out in the forest, root respiration was primarily influenced by photosynthesis and showed high-temperature sensitivity only in wet periods, heterotrophic respiration was also determined by soil temperature in wet and cold months, while by soil moisture content in warm and dry periods [28].

Among the sub-components of soil respiration, CO_2_ emissions from root respiration and CO_2_ emissions from the microbial decomposition of plant residues above the surface (litter production) are of outstanding importance. The amount of CO_2_ emissions resulting from the microbial decomposition of SOM should also be mentioned, which is found in root-free, undecomposed plant residue-free soil (basic respiration), in our case, in the No Input treatments. Root respiration is the respiration associated with root function, while the microbial decomposition of dead plant remains is respiration dependent on soil organic matter [5]. In the Síkfőkút research area, the soil respiration values of the different treatments converted to C content are shown in Table 1.

Our results show that the total soil respiration in the experimental area in the period 2002–2004 was 1.1956 g C/m^2^/day, while in the period 2010–2012 it was 1.6212 g C/m^2^/day. The year 2010 was extremely rainy, even the otherwise dry summer months were wet, which significantly increased the value of precipitation in that year and thus also in these three years. In the period between 2002 and 2004, the average moisture value of the soils of treatment C was 22.6%, while in the period 2010–2012 it was 27.4%. Presumably, this difference explains the discrepancy. Wetter soils increased the photosynthetic activity of vegetation, resulting in increased root respiration. The value of root respiration linked to plant activity in the two wet periods showed values of 0.2031 and 0.2399 C/m^2^/day, while the carbon amount of soil respiration resulting from the decomposition of the one-year litter cover showed values of 0.4345 and 0.5932 C/m^2^/day. Finally, the basic soil respiration, which comes from the soil without plant roots and dead plant parts (in our case, the NI treatment) was 0.9228 and 1.2366 C/m^2^/day.

According to some authors, as a result of the climate becoming warmer and drier, the net primary production decreases, as water is the limiting factor for plant growth, thereby reducing the amount of CO_2_ absorbed by the vegetation [29,30]. A warming climate reduces net primary production (NPP) more than respiration, so CO_2_ emissions from the soil may become more pronounced, thus drought causes soil carbon loss. The significant effects of persistent soil dryness on the components of soil respiration have already been reported in several studies [31,32].

In contrast, our results did not support the above finding. In our case, in the case of drier soils, CO_2_ emissions decreased significantly. Our results showed that in the wetter period of 2010–2012, the activity of the three investigated types of respiration increased compared to the drier soil measurements of 2002–2004. However, the root respiration is only 18.1%, the respiration resulting from the decomposition of fresh (younger than one year) litter is 36.5%, while the basic respiration, which shows the decomposition of the older carbon stock of the soil, is similarly 34%. These results show that soil respiration associated with the decomposing activity of microorganisms reacts much more sensitively to a decrease in soil moisture than root respiration associated with plants. This is because the plants can obtain the required amount of water from the deeper soil layers, so the moisture content of the upper 15 cm layer of the soil affects root respiration to a lesser extent.

The change in NPP is also significantly influenced by the rate of precipitation reduction. Based on surveys in dry forests [3], they experienced a much smaller net primary production and thus a smaller annual litter production. However, a much higher (2.5 times) carbon content was reported during the examination of the soils of dry forests. The above-mentioned reductions in different respiration types also support this.

According to some authors, the heterotrophic component of soil CO_2_ emissions is more sensitive to drought stress than the autotrophic component [33,34], while other studies have shown that the drought stress period mostly reduced the ratio of root and mycorrhizal respiration compared to the heterotrophic component from microbial respiration [35,36]. The reason for this contradictory finding may be the functional difference between the study area and vegetation types [37].

Within terrestrial ecosystems, forest areas are important due to their large extent and carbon storage capacity. However, in times of drought, they can act as a source of carbon, if the amount of carbon absorbed is less than the amount of carbon emitted, the consequence of which is an increase in atmospheric carbon dioxide concentration. Identifying the source of carbon loss from the soil is a significant task. An important question is what the proportion and extent to which the autotrophic and heterotrophic components contribute to CO_2_ emissions from the soil is.

## 3. Materials and Methods

### 3.1. Study Area

**Figure 8:**
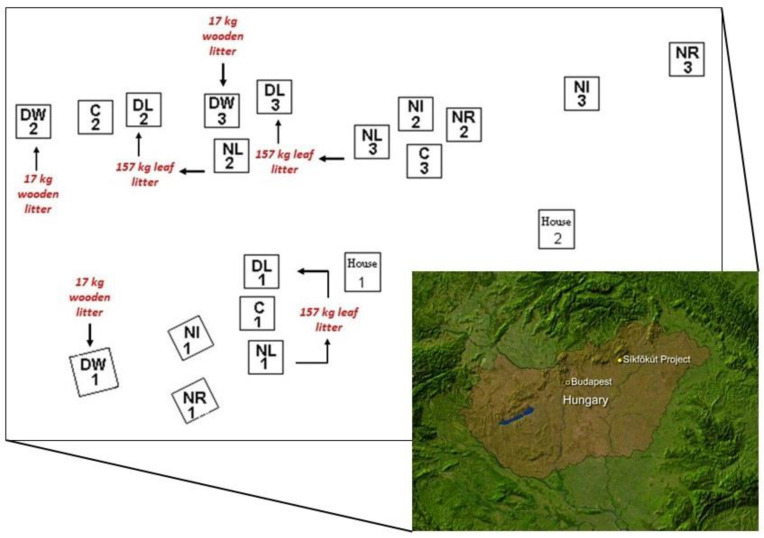
The Síkfőkút DIRT Project research area and layout of plots (Hungary).

The Síkfőkút DIRT Project research area is located in the southern, hilly landscape of Bükk, belonging to the Northern Central Mountains, northeast of Eger (Hungary) (Figure 8). Geographical coordinates of the project: 47°55′34″ N and 20°26′29″ E, altitude 320–340 m. According to the soil survey [38], the soils are Chromic Protovertic Luvisols (Clayic, Cutanic) and Protovertic Endostagnic Abruptic Luvisols (Clayic, Cutanic) [39]. The 27-hectare protected forest area is now under the supervision of the Bükki National Park. This research area is covered by the average 110-year *Quercetum petraeae-cerris* association with *Quercus petraea* and *Quercus cerris* species in the tree layer. The litter (organic matter) manipulation experimental plots were established in 2000 as part of the USA ILTER (International Long-Term Ecological Research) DIRT (Detritus Input and Removal Treatments) project. The DIRT plots in Síkfőkút were established according to the methods used in the USA DIRT Project [20]. In the case of the six treatments, three parallel plots were established, so a total of 6 × 3 = 18 experimental plots of 7 × 7 m (49 m^2^) were set up (Table 2).

### 3.2. Soil Sampling and Test Methods

Soil cores were collected from the 0–15 cm layers in mineral soil with a 20 mm diameter Pürckhauer 1175/1000 mm soil corer (Bürkle GmbH). In the process, we made an average sample from 5-point samples for each plot. These average samples were well homogenized and sieved through a 2 mm soil sieve. These average samples were used to determine the carbon (C) content and moisture content of the soil in three replications. Total C concentrations in the ground and homogenized soil samples from all three depths were determined using a combustion analyzer (VarioMax CN analyzer, ElementarAnalysensysteme GmbH, Hanau, Germany). To analyze carbonate C, replicate samples were heated in a muffle furnace at 450 °C for 16 h, then measured using the combustion [40]. The moisture content of the soil was determined using the traditional oven-dry method. The samples were dried in the laboratory at 105 °C until the mass was constant.

The soda lime (SL) method [41,42] was used to measure soil respiration (CO_2_ emissions). The measurements were done regularly, every month. We performed two measurements per plot, and also took a blind sample for every third plot. In this way, soil respiration was measured from a total of 3 × 2 (6) measurement points per treatment. A known weight of soda lime (Merck, granules with indicator, CO_2_ absorption capacity ≥25%) was placed in two open tins on each plot and each was covered with a plastic bucket. The lower rim of the buckets was buried 1–2 cm deep in the soil to prevent direct contact of the SL with the atmosphere. After 24 h, we collected the tins and measured their mass again under laboratory conditions. The amount of CO_2_ carbon absorbed by soda lime can be calculated from the two dry matter masses (before and after placement) with the help of the following formula:

(1) $$C\left(\mathrm{mg}\times m^{-2}\times h^{-1}\right)=\frac{\left(B-A-V\right)\times 1000\times 1.69\times 0.2729}{a\times t}$$

where:

A = dry weight before placement (g)

B = dry weight after placement (g)

V = average of blind measurements

a = area covered by the bucket (here 0.07122 m^2^)

t = absorption time (h)

The factors in the formula and their functions [42]:

1000—responsible for conversion between mg and g

1.69—Grogan’s correlation factor (eliminates the loss of crystal water in soda lime during drying)

0.2729—the ratio of the carbon content of CO_2_.

The results of the plots with the same treatment were averaged and compared with the soil moisture data. The determination of the subcomponents of soil respiration was carried out using Nadelhoffer et al.’s [20] method. Based on this, the carbon from the decomposition of one year’s litter cover is equal to the average difference between the C and NL plots and the difference between the DL and C plots. Root respiration corresponds to the difference in CO_2_ emissions between C and NR treatments.

### 3.3. Statistical Methods

Statistical analyses were performed using ANOVA and Tukey’s HSD test (comparing both moisture values of treatments and soil respiration values). The correlation between moisture and CO_2_ emissions was tested by analysis of variance.

## 4. Conclusions

The autotrophic (respiration of plant roots) and heterotrophic (derived from microbial decomposition) components of soil respiration react differently to different environmental factors and contribute to the total carbon turnover of soils to varying degrees. Therefore, their isolation and determination of their contribution to total soil respiration are essential for understanding soil carbon loss. Our results show that the decreasing litter production (and, in parallel, also the root respiration, because it is presumably directly proportional to the net primary production), results in a decrease in the heterotrophic respiration related to the decomposing processes, which results in a slower decomposition of soil organic matter. Based on our studies, the increase in the intensity of root respiration in wetter soils is only half of the respiratory increase associated with the decomposing activity. As a result of the drastic removal of litter, the carbon content of the soil decreased. Furthermore, an interesting exception for the soils of the No Litter and No Input treatments is that the drier microclimate (more intense evaporation due to the lack of a litter cover) and the loss of soil organic carbon together caused more intense drying of the soils, and the accompanying fracturing increased the loss of carbon in the deeper, wetter layers of the soils during the summer. Because of these processes, the efflux of CO_2_ from the soil increased. Together, these effects can significantly increase the loss of soil organic matter, which can lead to a large deterioration of soil quality. Some of these processes are natural processes, to which human activity, inappropriate land use, and climate change can contribute to a large extent. Based on our results, we can therefore recommend a reasonable soil use that minimizes water loss (by continuously covering the soil surface) and excessive disturbance (thereby reducing excessive aeration, which can increase microbial activity at deeper levels as well).

## Figures and Tables

**Table 1:** CO_2_ release expressed in C content ± SE values of soil respiration in the experimental plots in the dry and wet periods between 2002 and 2004 and 2010 and 2012 (*n* = 6). C = control; DL = Double Litter; NL = No Litter; DW = Double Wood; NR = No Roots; NI = No Inputs.

	g C/m^2^/day	2002–2004		2010–2012	
Treatments		Dry	Humid	Dry	Humid
C		1.23 ± 0.07	1.19 ± 0.06	1.31 ± 0.06	1.62 ± 0.03
NL		1.32 ± 0.06	0.94 ± 0.07	1.78 ± 0.07	1.17 ± 0.09
DL		1.49 ± 0.01	1.55 ± 0.04	1.26 ± 0.06	1.90 ± 0.02
NR		2.05 ± 0.11	0.99 ± 0.10	1.37 ± 0.09	1.38 ± 0.04
DW		1.29 ± 0.09	1.30 ± 003	1.35 ± 0.09	1.49 ± 0.02
NI		1.81 ± 0.01	0.92 ± 0.04	1.95 ± 0.05	1.23 ± 0.04

**Table 2:** The applied DIRT (Detritus Input and Removal Treatments) at the Síkfőkút Project site (Hungary) as part of the ILTER (International Long-Term Experimental Research).

Treatments	Description
Control (C)	Normal litter inputs. Average litter amount typical of the forest site.
No Litter (NL)	Aboveground inputs are excluded from plots. Leaf litter was removed by raking. This process was repeated continuously every year.
No Roots (NR)	Plant roots are excluded, there are no herbs above the ground, and growth from the side is also inhibited. The leaf litter is left on the ground. The plots were trenched around 100 cm deep with root-proof Delta MS 500 PE foil, which was 0.6 mm thick. To eliminate root production, plants were cleared (bushes had been cut out at the establishment).
No Inputs (NI)	Aboveground inputs are excluded from plots; the belowground inputs are provided as in NR plots. This treatment is a combination of NR + NL plots.
Double Wood (DW)	Aboveground wood debris inputs are doubled by adding wood to each plot. Annual wood litter amount was measured by boxes placed at the site, and double its amount was applied in the case of every DW plot.
Double Litter (DL)	Aboveground leaf inputs are doubled by adding litter removed from No Litter plots.
